# Genome-Wide Association Study in an Admixed Case Series Reveals *IL12A* as a New Candidate in Behçet Disease

**DOI:** 10.1371/journal.pone.0119085

**Published:** 2015-03-23

**Authors:** Jasper H. Kappen, Carolina Medina-Gomez, P. Martin van Hagen, Lisette Stolk, Karol Estrada, Fernando Rivadeneira, Andre G. Uitterlinden, Miles R. Stanford, Eldat Ben-Chetrit, Graham R. Wallace, Merih Soylu, Jan A.M. van Laar

**Affiliations:** 1 Internal medicine, Section immunology, Erasmus Medical Center, Rotterdam, The Netherlands; 2 Department of Internal Medicine, Erasmus Medical Center, Rotterdam, The Netherlands; 3 Department of Immunology, Erasmus Medical Center, Rotterdam, The Netherlands; 4 Department of epidemiology, Erasmus Medical Center, Rotterdam, The Netherlands; 5 The Generation R Study Group, Erasmus M Medical Center C, Rotterdam, The Netherlands; 6 Department of Ophthalmology, King’s College, London, United Kingdom; 7 Centre for Translational Inflammation, University of Birmingham, Birmingham, United Kingdom; 8 Department of Medicine, Hadassah-Hebrew University Medical Center, Jerusalem, Israel; 9 Department of Ophthalmology, University of Cukurova, School of Medicine, Adana, Turkey; University of Newcastle, AUSTRALIA

## Abstract

**Introduction:**

The etiology of Behçet’s disease (BD) is unknown, but widely considered an excessive T-cell mediated inflammatory response in a genetically susceptible host. Recent genome-wide association studies (GWAS) have shown limited number of novel loci-associations. The rarity and unequal distribution of the disease prevalence amongst different ethnic backgrounds have hampered the use of GWAS in cohorts of mixed ethnicity and sufficient sample size. However, novel statistical approaches have now enabled GWAS in admixed cohorts.

**Methods:**

We ran a GWAS on 336 BD cases and 5,843 controls. The cases consisted of Western Europeans, Middle Eastern and Turkish individuals. Participants from the Generation R study, a multiethnic birth cohort in Rotterdam, The Netherlands were used as controls. All samples were genotyped and data was combined. Linear regression models were corrected for population stratification using Genomic Principal Components and Linear Mixed Modelling. Meta-analysis was performed on selected results previously published.

**Results:**

We identified SNPs associated at genome-wide significant level mapping to the 6p21.33 (HLA) region. In addition to this known signal two potential novel associations on chromosomes 6 and 18 were identified, yet with low minor allele frequencies. Extended meta-analysis reveal a GWS association with the *IL12A* variant rs17810546 on chromosome 3.

**Discussion:**

We demonstrate that new statistical techniques enable GWAS analyses in a limited sized cohort of mixed ethnicity. After implementation, we confirmed the central role of the HLA region in the disease and identified new regions of interest. Moreover, we validated the association of a variant in the *IL2A* gene by meta-analysis with previous work. These findings enhance our knowledge of genetic associations and BD, and provide further justification for pursuing collective initiatives in genetic studies given the low prevalence of this and other rare diseases.

## Introduction

Behçet’s disease (BD) is a systemic auto-inflammatory occlusive vasculitis presenting with oral and genital aphthous ulcers, skin lesions, ocular inflammation and a pathognomonic pathergy test [1–3]. Although the etiology of the disease is largely unknown, it is considered to be an excessive inflammatory response possibly triggered by an infectious antigen in a genetically susceptible host. Prevalence varies from 110–420 per 100,000 in the Middle East (Turkey) to about 2 per 100,000 in Western countries [3,4]. In patients of Turkish origin there is a positive family history in 12% of the cases with a sibling risk ratio between 11–52 [3,4]. The heritability of BD has been estimated to range between 20 to 60%, with the strongest genetic association embracing variants in HLA-B51, explaining about 20% of the disease heritability [3,4]. The combination of epidemiological and genetic data suggest causal involvement of both genetic and environmental factors [5].

Over the years, numerous single nucleotide polymorphisms (SNPs) have been found associated with BD [6–9]. In 2010 the first genome-wide association study (GWAS) in BD cohorts of Turkish and Japanese origin demonstrated association of various variants in the known HLA-B51 domain, and two new association signals, mapping to the interleukin-10 (*IL10*), and the IL-23 receptor–IL-12 receptor Beta2 (*IL23R–IL12RB2*) locus [10,11]. These associations later have been confirmed in an Iranian cohort [12]. Yet another study in Algerian individuals only replicated the associations from the IL10 variants [13]. GWAS in Chinese and Korean individuals reported associations of SNPs mapping to two other loci, with regions containing the signal transducer and activator of transcription 4 (*STAT4*) and GTPase of immune associated protein *GIMAP* genes, respectively. Polymorphisms in *IL10* and *IL23R–IL12RB2* were not found to be significantly associated with BD in neither of those studies (14,15). More recently, associations with *CCR1*, *STAT4* and *KLRC4* were identified by means of imputation and GWAS meta-analysis [14]. This study also identified the *IL12A* region as suggestively associated with BD, but genome wide significance (GWS) was not reached. Discrepant association results, as those observed for variants mapping to the *GIMAP* locus, might be explained by the different ethnic origin of the cohorts studied [15,16]. Therefore, studying cohorts of diverse ethnic background may be useful to understand the origin of these differences. Moreover, the inclusion of multiple ethnicities results in larger datasets (representing higher power) crucial for these analyses given the typically small effects of common genetic variants discovered by the GWAS approach [17]. Nonetheless, GWAS results can be confounded by ancestry differences between cases and controls, potentially leading to spurious associations. This challenge in multi-ethnic studies has so far been approached by methods that take into account confounding by genetic ancestry, such as correction for Genomic Principal Components (GPC) [18]. More recently, linear mixed models (LMM) have been introduced as a reliable method to correct not only for ancestry differences, but also for family structure and/or cryptic relationships [19].

We have therefore set up a multi-center GWAS including BD patients of both Middle Eastern and Western background. We demonstrate that by means of novel statistic approaches it is feasible to run a GWAS in a small multiethnic cohort and use these results for meta-analysis.

## Materials and Methods

### Study populations

A total of 369 unrelated BD patients from 18 different geographic origins were included in our study (Table 1). The age range was 18 to 73 years (mean 47 years). All patients fulfilled the International Study Group (ISG) criteria of BD diagnoses [20]. No exclusion criteria were applied.

**Table 1:** Cases before and after QC.

Ethnicity	Number of patients	Collection	Number of patients after QC
Afghanistan	1	[1]	1
Iranian	4	[1]	4
Lebanese	1	[1]	1
Cape Verde	2	[1]	2
Curacao	1	[1]	1
Dominican Republic	1	[1]	1
Dutch caucasian	24	[1]	20
Greece	1	[1]	1
Israel	1	[1]	1
Jordan	1	[1]	1
Morocco	16	[1]	12
Surinam	2	[1]	1
Thailand	1	[1]	1
China	1	[1]	1
Turkey	35	[1]	32
Turkey	91	[2]	87
Arab Jerusalem ancestry	110	[3]	98
UK caucasian	38	[4]	33
Syrian	38	[5]	38
			
**Total**	**369**		**336**

Collection at [1] Erasmus MC at Rotterdam, The Netherlands, [2] University Hospital of Cukurova, Turkey, [3] St John’s Ophthalmic Hospital, Jerusalem, Israel, [4] St Thomas’ Hospital, London, UK, [5] University Hospital, Damascus, Syria, Ethnicity of cases after QC.

As controls, 87 Syrian healthy volunteers (22–73 years, mean 51 years) and 5,756 participants from the Generation R study, a multi-ethnic birth cohort in Rotterdam (4–9 years, mean 6 years), The Netherlands, were used [21].

Replication was pursued in a cohort of 82 BD patients who met the ISG criteria, and 98 ethnically matched controls in Western European, collected from Birmingham and Midland Eye Centre, Birmingham, UK.

The ethics committees of the involved centers (Erasmus MC (METC), Ethical Committe at Çukurova University and Sandwell and West Birmingham Hospitals Trust Ethics Committee) approved the study, and written informed consent was obtained from all participants.

### Genome wide genotyping

Genotyping of cases and controls (including the Generation R Study) was performed using the Illumina HumanHap 610K and/or 660 K arrays, following manufacturers protocols. Quality Control (QC) was performed individually for each set following a standard protocol to exclude samples and SNPs with low quality genotyping. Markers with minor allele frequency (MAF) ≤ 1%, missing genotypes ≥5% or which failed an exact test of Hardy-Weinberg equilibrium proportions in the controls (P<1x10–7) were excluded from the datasets. Samples with gender discrepancy, excess of heterozygosity, duplicates or samples with relatedness or other inconsistencies were also removed. In a subsequent stage, we extracted SNPs common to both platforms which passed individual QC and then applied the QC protocol for the combined dataset.

### Phenotype-Genotype Analyses

#### Heritability

To characterize the extent to which common genetic variants play a role in BD susceptibility, we applied a recently proposed approach for estimating heritability based on genome-wide sharing between distantly related individuals, as implemented in the GCTA software in combination with the determination of ancestry-aware kinship coefficients by REAP software [22,23]. No pairs exceeded the standard cutoff coefficient of 0.025 for genetic relatedness, confirming that no two individuals in the analysis were closer than third degree cousins. Relatedness coefficients calculated from REAP were used in the relatedness matrix before implementing GCTA. A disease prevalence of 0.42% was used in the modelling [3,4].

#### Genome-wide association analysis

Association between BD susceptibility and 553.224 genome-wide SNPs was carried out using a regression framework adjusting for population stratification. GWS threshold for the association was established at p<5×10^-8^. Two different strategies were followed to adjust for population sub-structure in the data.

#### Genomic Principal Components

In the first approach, 20 GPC were obtained for all pairs of individuals in the combined dataset based on the similarities of individual genotypic profiles for 37,060 independent autosomal SNPs contained in the HapMap Phase II release 22 build 36 [24] using Multi-Dimensional Scaling (MDS) as implemented in PLINK (http://pngu.mgh.harvard.edu/purcell/plink/). Afterwards, we performed logistic regression adjusting for those 20 GPC.

#### Linear Mixed models

In the second approach we used LMM in order to account for possible population structure in the association analysis. This methodology estimates the level of relatedness even between independent individuals using the genotyped markers. By modeling the dissimilarity between genotypes of the subjects, it is able to correct the association results for stratification. This approach is implemented in the publicly available EMMA eXpedited (EMMAX) software [25]. Since the effect sizes reported by EMMAX are based on *Mantel*–Haenszel statistics, the odd ratios for associations of the significant SNPs were estimated from the regular logistic regression models performed in PLINK.

#### Meta-analysis

Meta-analysis of our own study results with published results for the BD suggestive associated SNP rs17810546 [14] was performed using an inverse variance fixed-effects approach in METAL [26]

### Replication analysis

Four SNPs: rs8187722, rs439033, rs11969661 and rs17087141 were genotyped using TaqMan assays designed by Applied Biosystems, Warrington, UK in 82 BD patients and 98 control samples. In short, PCR was performed in 384-well plates with a 10 μl total reaction volume containing 20 pg/ml DNA (samples) or water (controls) and 2x LC-480 probe master (Roche, West Sussex UK) followed by endpoint genotyping analysis using the LC-480 system (Roche, West Sussex UK). Genotypes were determined as either homozygous (e.g. AA or GG) or heterozygous (e.g. AG) according to the presence or absence of fluorescence for each genotype.

## Results

An overview of the ethnicity of the included BD cases is presented in Table 1; all controls were collected in St John’s Ophthalmic Hospital, Jerusalem and had Arab ancestry. In order to gain power and match all remaining ethnicities, this dataset was combined with the genotyped samples from the multiethnic Generation R Study.

### Genome wide Genotyping

Genotyping was performed in 456 samples (369 cases and 87 controls) collected from the different participating hospitals (Erasmus MC at Rotterdam, The Netherlands, University Hospital of Cukurova, Turkey, St John’s Ophthalmic Hospital, Jerusalem, Israel and by St Thomas’ Hospital, London, UK). After quality control, 553,224 genotyped SNPs in 423 of these samples remained. We excluded 33 patients (including 1 control) because of low Illumina call rate (< 97.5%) and one more individual was excluded for gender mismatch. After QC of this combined dataset, 336 cases and 5843 controls were available for analysis.

### Phenotype Genotype Analyses

#### Heritability

The heritability estimate, based on the 505,454 genotyped SNPs and 4,855 unrelated individuals (296 cases) was 32.0% (95%CI 21.0–44.0%;P = 5.5E-17). Thus a considerable percent of the BD risk is explained by the additive effect of the common SNPs analyzed.

#### Genomic Principal Components

**Fig 1:**
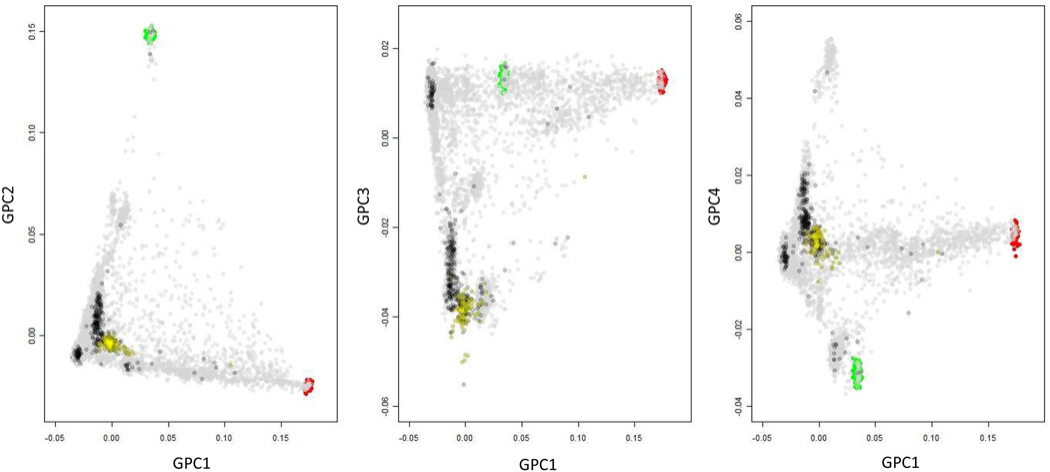
Genetic substructure of the Combined GWAS dataset. Two-dimensional scatterplots from multidimensional scaling analyses of the Generation R Study and Behçet collected data together with the three initial Panels form the HapMap Project. Each dot represents an individual in the dataset. Color codes: Grey = Generation R, Black = Behcet Cases, Yellow = Jordan controls. Blue = CEU, Red = YRB, Green = JPT.

The representation of the first GPC for the combined dataset projected together with the three reference panels of the International HapMap Project Phase II known as YRI, CEU and CHB/JPT representing populations of African, European and Asian background respectively [27] is shown in Fig. 1. As shown by the overlapping distribution across the four first GPC, BD cases (represented in black) are well matched by genetic ancestry to controls (represented in yellow Syrian volunteers and grey for Generation R participants).

#### Genome wide association analysis

**Fig 2:**
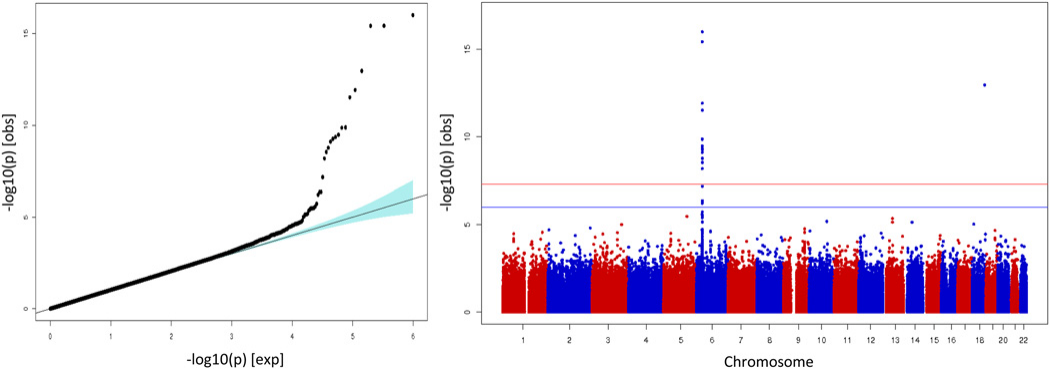
Behçet GWAS results using Genomic Principal Components (GPC) adjustment. Each dot represents an SNP in the dataset. QQ-plot (left). Associated SNPs deviating from the null hypothesis of no association (identity line). Manhattan plot (right). SNPs showing association with the disease map to chromosome 6 and a singleton in chromosome 18.

Association analyses were performed for 505,454 SNPs. As expected in such structured population, results from analysis without any type of stratification adjustment, showed association through the whole genome and a Genomic Inflation Factor (λ) of 5.25 (S1 Fig.). After GPC correction, the association analysis showed minimal inflation of the test statistics with a λ of 1.035 (Fig. 2). The top associated SNPs mapped to the HLA region on chromosome 6 with rs7770216 being the most significant (Table 2). Yet, another GWS signal in chromosome 18 driven by rs17087141 mapped to a region containing the uncharacterized miscRNA LOC400655.

**Table 2:** GWS SNPs associated with Behçet’s disease susceptibility using GPC and EMMAX approaches.

							20 PCs		EMMAX*
CHR	SNP	BP	A1	A2	MAF	N	OR	P	P
6	rs9380215	31157634	A	G	0.09	6178	2.132	1.32E-10	3.15E-15
6	rs4947296	31166157	C	T	0.09	6178	2.131	1.38E-10	4.98E-15
6	rs9263509	31174398	T	C	0.16	6179	1.82	6.62E-09	2.69E-10
6	rs2233984	31187243	A	G	0.09	6179	2.064	3.35E-10	1.08E-15
6	rs4495304	31188697	C	T	0.09	6028	2.069	7.92E-10	3.84E-14
6	rs4959053	31207556	A	G	0.09	6175	2.348	1.20E-12	3.90E-17
6	*rs2523589*	*31435313*	*C*	*A*	*0.43*	*6177*	*0.5466*	*2.92E-09*	5.83E-08
6	rs2844575	31442924	G	A	0.48	6173	1.81	5.62E-10	5.92E-08
6	rs9266409	31444547	C	T	0.25	6178	2.183	3.66E-16	2.62E-18
6	rs2253907	31444849	A	G	0.48	6175	1.797	4.41E-10	1.46E-09
6	rs7770216	31448590	T	G	0.26	6072	2.198	9.75E-17	2.25E-18
6	rs6933050	31451611	C	T	0.25	6178	2.183	3.69E-16	2.59E-18
6	rs1131896	31487094	A	G	0.31	6141	1.647	6.86E-08	2.66E-09
6	rs2256028	31487177	T	G	0.21	6177	1.784	1.72E-09	7.49E-12
6	rs2848713	31492458	A	G	0.1	6173	2.118	3.03E-12	7.08E-22
*6*	*rs11969661*	*160626409*	*T*	*C*	*0.02*	*6179*	*2.696*	*1.30E-03*	*5.15E-09*
*6*	*rs439033*	*160739406*	*C*	*T*	*0.02*	*6163*	*2.016*	*2.30E-03*	*1.14E-08*
*6*	*rs8187722*	*160784748*	*G*	*A*	*0.01*	*6179*	*1.696*	*1.00E-04*	*2.81E-15*
6	rs17087141	69036707	C	T	0.03	6167	4.925	1.08E-13	2.81E-18

Results for the association analysis when correcting for 20 PCs to adjust for stratification. In italic font the SNPs which show association only by one of the two approaches.

* Odd ratios cannot be calculated by EMMAX approach.

Additional association analyses using different strategies including reduction of number of matching controls (to different case/control ratios) and incorporating different number of genomic principal components in the models yielded similar results for effect coefficients and genomic inflation factors (data not shown).

**Fig 3:**
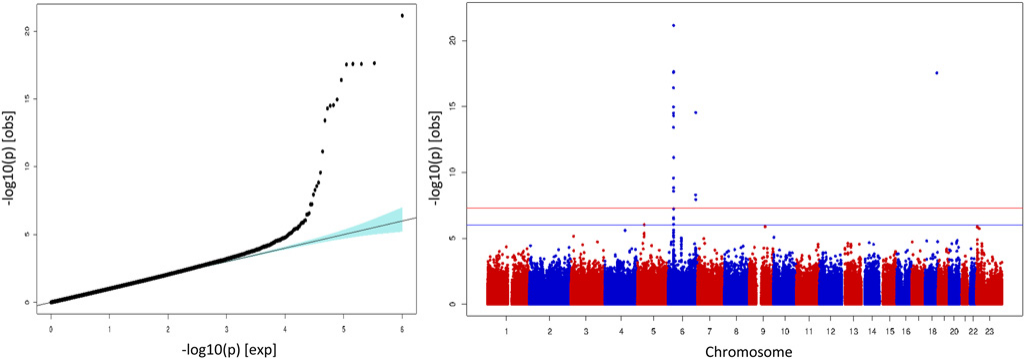
Behcet GWAS results using Linear Mixed Models Genomic approach. Each dot represents an SNP in the dataset. QQ-plot (left). Associated SNPs deviating from the null hypothesis of no association (identity line). Manhattan plot (right). SNPs showing association with the disease map to two different signals in chromosome 6 and a singleton in chromosome 18.

Results from the GWAS analysis using EMMAX yielded three independent GWS signals (Table 2). In addition to those mapping to the two previously described BD loci from chromosomes 6 and 18, an additional signal was found on chromosome 6q25.3 6 in the solute carrier family 22 member 3 (*SLC22A3*) gene region. The three most significant markers underlying this GWAS signal included rs11969661, rs439033 and rs8187722 (Table 2). Using EMMAX resulted in adequate adjustment for population stratification as corroborated by λ = 1.0 (Table 2) and the absence of early deviation from the identity line in the QQplot (Fig. 3).

**Fig 4:**
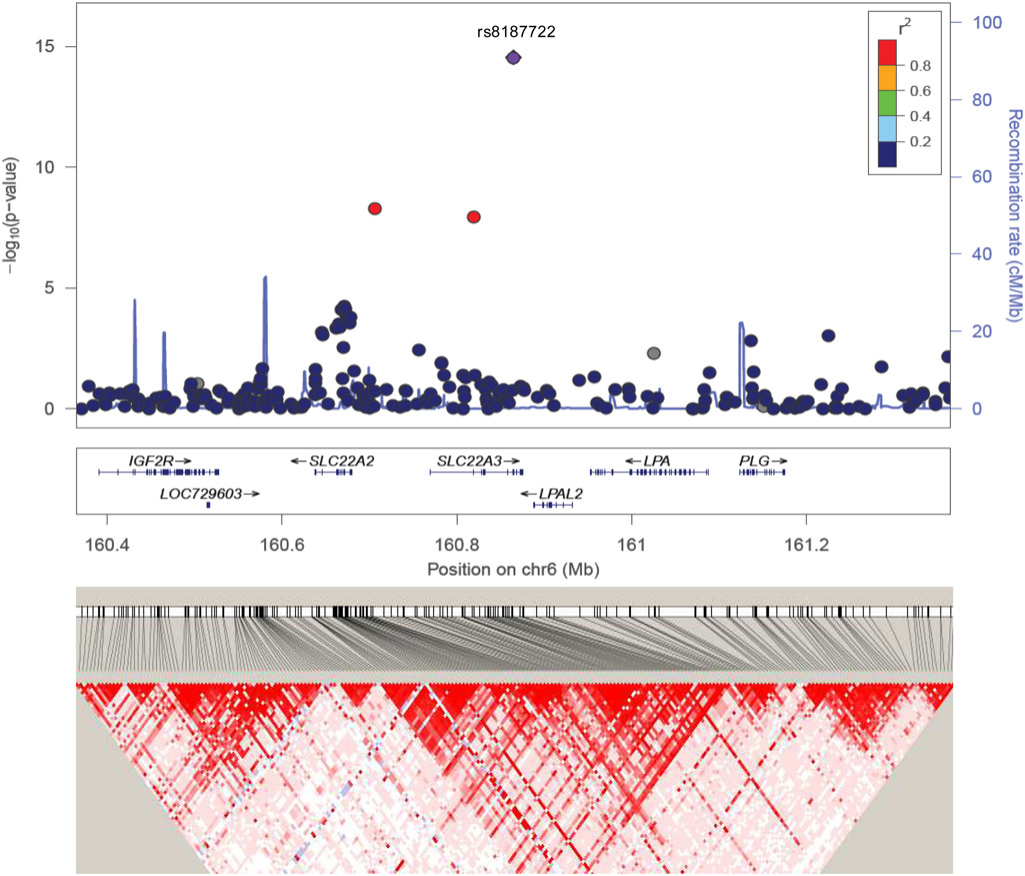
SNP association plot for Behçet’s susceptibility-associated region of chromosome 6q25.3. Dots represent GWAS P-values (EMMAX approach) and positions of SNPs found within the 6q25.3 locus. The top SNP, i.e. rs8187722, is denoted by a diamond. Different colours indicate varying degrees of pair-wise linkage disequilibrium (1000 Genomes Nov 2010 CEU) between the top SNP and all other genotyped SNPs. Genetic coordinates are per 1000 Genomes Nov 2010-CEU. Bottom, LD heat map based on D’ values from the combined population under study including all SNPs in the 500Kb region.

**Fig 5:**
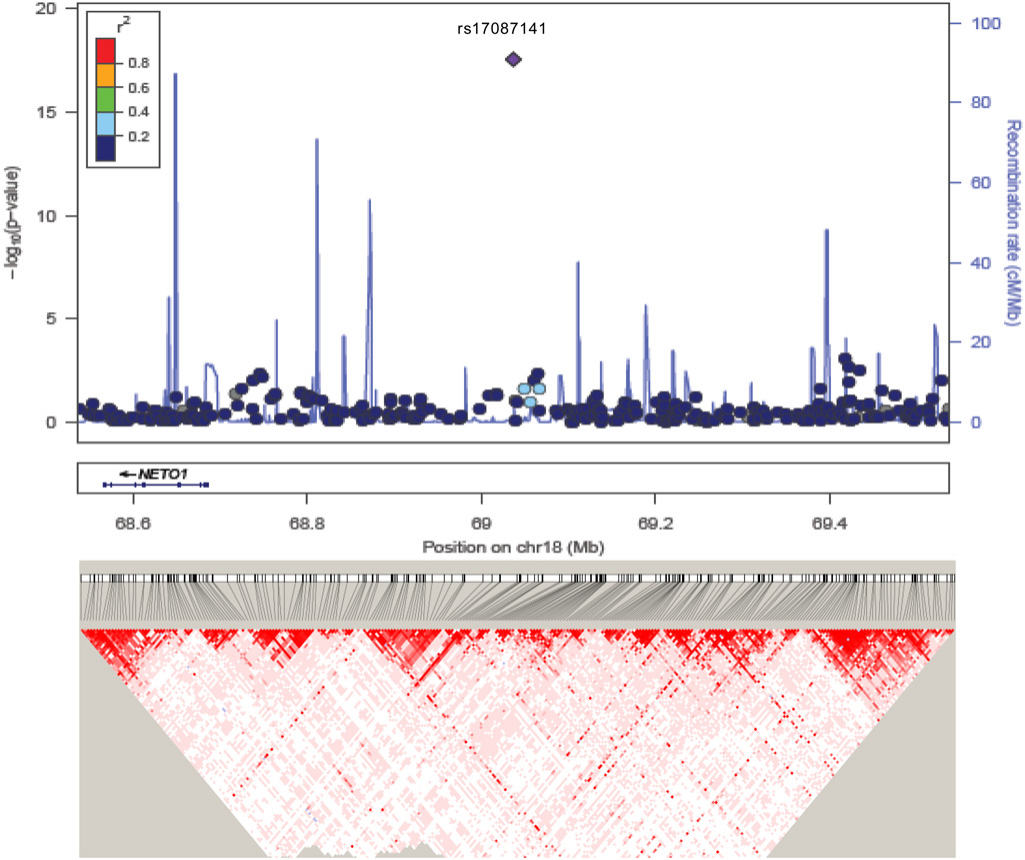
SNP association plot for Behçet’s susceptibility-associated region of Chromosome 18q22.3. Dots represent GWAS P-values (EMMAX approach) and positions of SNPs found within the 18q22.3 locus. The top SNP, i.e. rs17087141, is denoted by a diamond. Different colours indicate varying degrees of pair-wise linkage disequilibrium (1000Genomes CEU) between the top SNP and all other genotyped SNPs. Genetic coordinates are per 1000 Genomes Nov 2010-CEU. Bottom, LD heat map based on D’ values from the combined population under study including all SNPs in the 500Kb region.

The most significant SNP identified by the EMMAX approach maps also to the HLA region in chromosome 6. The regional association plot of this HLA-B region is shown in S2 Fig. This HLA-B region has been associated with BD susceptibility in previous GWAS analyses [10,22]. Figs. 4 and 5 show the association plots for the other two GWS signals in 6q25.3 (*SCLA22A3* locus) and 18q22.3, which have not been previously reported as associated with BD.

### Meta-analyses

By scanning published BD GWAS, we identified a variant suggestive of association (P = 6.01x10–7) [14], showing strong evidence for association with BD in our study, although not at GWS level. After meta-analysis of our results with those published by Kirino et al. in two Turkish cohorts the leading SNP (rs17810546) in *IL12A* reached GWS. The G-allele conferred a 1.7 increased risk for BD (OR = 1.66; 95%CI [1.42,1.93]; P = 1.12E-10 (Table 3.)).

**Table 3:** Meta-analysis of leading SNP in IL12A.

Study	N	N cases	OR	CI 95%	P value
Turkish replication*	2487	1209	1.64	[1.31 – 2.04]	1.20E-05
Turkish discovery*	1447	821	1.41	[1.06 – 1.89]	2.00E-02
Current study	6179	336	2.06	[1.49 – 2.84]	9.31E-06
Combined	10113	2366	1.66	[1.42 – 1.93]	1.12E-10

Association results for rs17810456 (G-allele) in two Turkish cohorts (14) and the current study.

* From Table 1 Kirino et al. 2013

### Replication

No significant evidence for replication of the leading SNPs from the GWAS signals of chromosome 6 and 18 was found, while the MAF of the genotyped markers were very similar to those reported in healthy individuals.

## Discussion

Enabled by innovative novel methodology we could run a GWAS in a rare condition like BD within a unique case collection of multiethnic background. We identified variants associated with BD mapping to the well-established MICA-HLA-B locus and to two regions on chromosome 6 in the *SLC22A* gene region and on chromosome 18 in an uncharacterized region. Moreover, meta-analysis with previous published results enabled the identification of a GWS association with variants in the *IL12A* region. All together, these common variants across this four loci explained up to 32% of the variance in BD risk.

Methods for the calculation of heritability estimates from SNP microarray data of population-base studies have recently emerged [22,23]. We could determine that the narrow sense heritability of BD explained by common variants in our study was 32%, estimate in line with those obtained by previous reports based on family data [3,4,28], albeit smaller. This could be explained by the overestimation of heritability estimates in family-based design, as a result of biases due to epistatic interactions or shared environment. In addition, GCTA estimates should be interpreted as the lower bound of the true additive genomic influence on heritability, since the genetic variance is limited to the common SNPs present in the arrays[29]. Therefore variance explained by rarer variants (MAF<0.01) and/or causal variants that were not genotyped or are not tagged by the SNPs on the genotyping array will be missed. As GCTA is intended for studies on homogeneous populations it is necessary to use appropriate methods to calculate the relatedness matrix in studies with admixed individuals to avoid overestimation of the heritability. Therefore, to obtain unbiased estimates we used REAP for the estimation of the kinship coefficient [30].

Methods based on LMM enabled the analysis of GWAS data in a cohort of mixed ethnicity of relative small sample size, maximizing power. The fact that our GWAS identified well-established variants in the HLA-B locus, provides certainty that the methodological approach was sound and in fact applicable to other rare diseases. Expanding the current case collection or performing meta-analysis with other collections is warranted. Efforts including datasets of different ancestries, should be analyzed with methodologies which take into account different genomic architecture based on ethnic background. Such methods can also be applied at the meta-analysis level as implemented in MANTRA [31].

In addition to the signal in the well-known HLA locus on chromosome 6, we also identified two genomic regions of interest. Nevertheless, all variants had relatively low MAF (2–3%) and thus their significance should be interpreted with caution. We could not find evidence for replication of the associations in an independent yet underpowered case/control set. Considering the low MAF and the very limited power of the replication further scrutiny in an expanded dataset is required to confirm these variants as real or spurious associations.

Yet another GWS association with rs17810546 in the *IL12A* locus was identified by meta-analyses of our results with those reported previously in the literature. These results support the role of this locus in the susceptibility of BD. Kirino et al., described this locus as suggestive for association with BD. In that study the variants did not were reach GWS in the combined analysis likely due to the variant being monomorphic in the samples of Japanese origin included in the study. Variants in *CCR1-CCR3*, *STAT4* and *KLRC4* which were polymorphic in both Turkish and Japanese populations surpassed GWS thresholds, suggesting that the lower power likely contributed to the non-significant findings in the *IL12A* locus.

BD is a rare disease, even in countries with the highest prevalence. Collecting the patient material for large multi-ethnic genetic studies proved to be challenging. Not surprisingly, the first two GWAS studies reported in BD were in two separate cohorts with a single ethnicity, a Turkish and a Japanese cohort [10,11]. After meta-analyses of the data of both studies, polymorphisms in *IL10* and *IL23R–IL12RB2* proved to be associated at GWS level. In our study, these two polymorphisms were not associated with BD, nor in two other recent studies in Chinese and Korean individuals [32,33]. Moreover, the *GIMAP* variants presented in the Chinese and a Korean cohort were not identified in the other studies. Recently, Ortiz-Fernandez et al., reported no association of *GIMAP* variants in a Spanish study but also acknowledged lack of power as a likely cause for this negative results [16]. Considering that most of our cases are of admixed ethnicity differences in ethnic background, specific pathology or clinical features of the cases are a more plausible explanation for the lack of replication of those variants in our study on top of limited power [32,33].

In conclusion we have shown that appropriate methodological approaches enable performing GWAS in cohorts of admixed ethnicity, combining cases of different ethnic origin as is often required in rare conditions like BD. As such, previously reported SNPs in the MICA-HLA-B locus reached GWS in the current approach. Additionally, we have shown that results of a mixed cohort can successfully be used in meta-analysis, by which we could establish the involvement of previously putative variant in the *IL12A* locus in the susceptibility of the disease.

Our results emphasize the need of collaborative efforts intending to enlarge the collection of BD cases, which can enable a well-powered setting for detection of genetic associations with the disease, especially when interrogating low-frequency variants.

Potentially novel loci need to be further explored in additional studies, and ultimately coupled with functional studies, allowing a better understanding of the pathophysiology and the genetic architecture underlying the risk for BD.

## Supporting Information

S1 Fig Behçet GWAS results using not adjustment for population stratification. Each dot represents an SNP in the dataset. **QQ-plot (left)**. Associated SNPs deviating from the null hypothesis of no association (identity line) evidence high inflation. **Manhattan plot (right)**. SNPs though all chromosomes show association with the disease.
(TIF)

S2 Fig HLA Regional Association Plot. GWAS results forBehcet susceptibility using Linear Mixed Models approach.
Top SNP rs2848713 EMMAX approach (MAF = 0.10, A-risk allele) is denoted by a diamond. Marker rs7770216 is the top SNP for the Genomic Principal Components adjustment approach (MAF = 0.25, T-risk allele). Marker rs4495304 is the top SNP in the association reported by Mizuki et al. (MAF = 0.09, A-risk allele) for the GWAS of Behçet susceptibility. Bottom, LD heat map based on D’ values from the combined population under study including all SNPs in the 500Kb region.
(TIF)
