# Supplementary figures and images for: Genome-Wide Association Study in an Admixed Case Series Reveals IL12A as a New Candidate in Behçet Disease
Source: PLoS One. 2015 Mar 23;10(3):e0119085. doi: 10.1371/journal.pone.0119085 (PMC4370488; doi:10.1371/journal.pone.0119085)

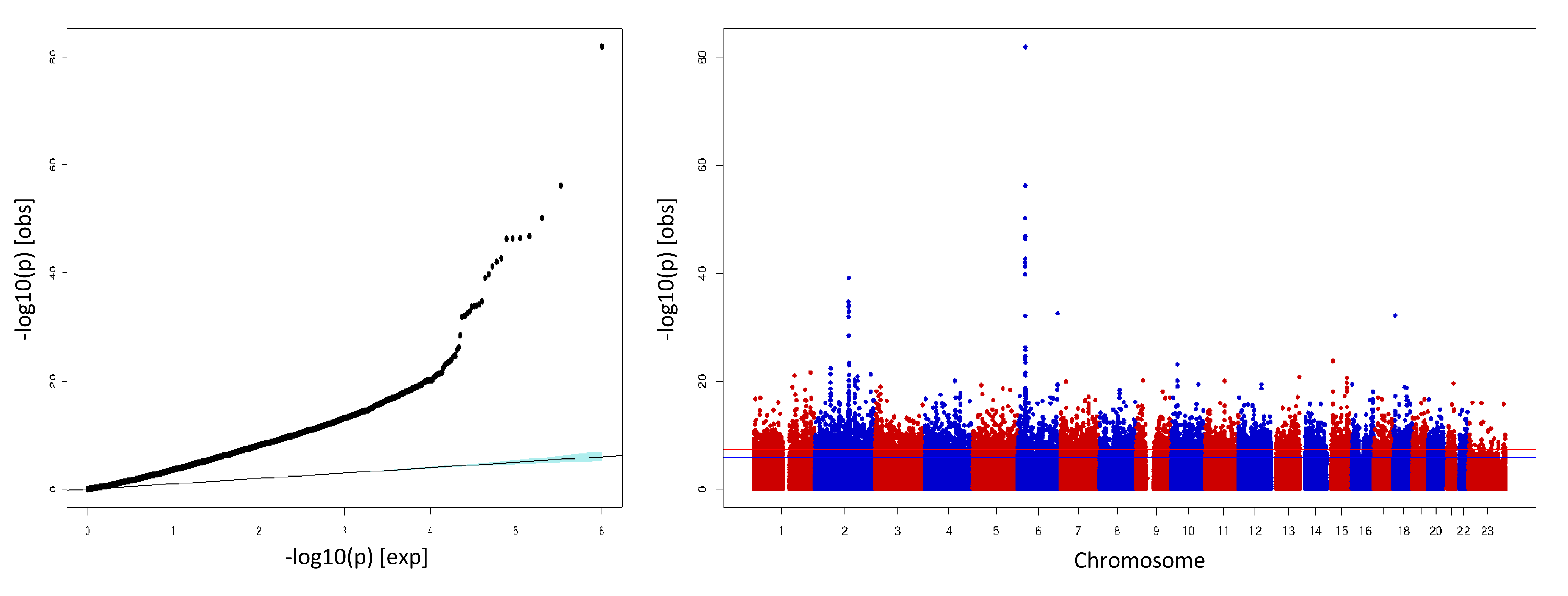

Supplement: S1 Fig — Each dot represents an SNP in the dataset. QQ-plot (left). Associated SNPs deviating from the null hypothesis of no association (identity line) evidence high inflation. Manhattan plot (right). SNPs though all chromosomes show association with the disease. (TIF) [file pone.0119085.s001.tif]

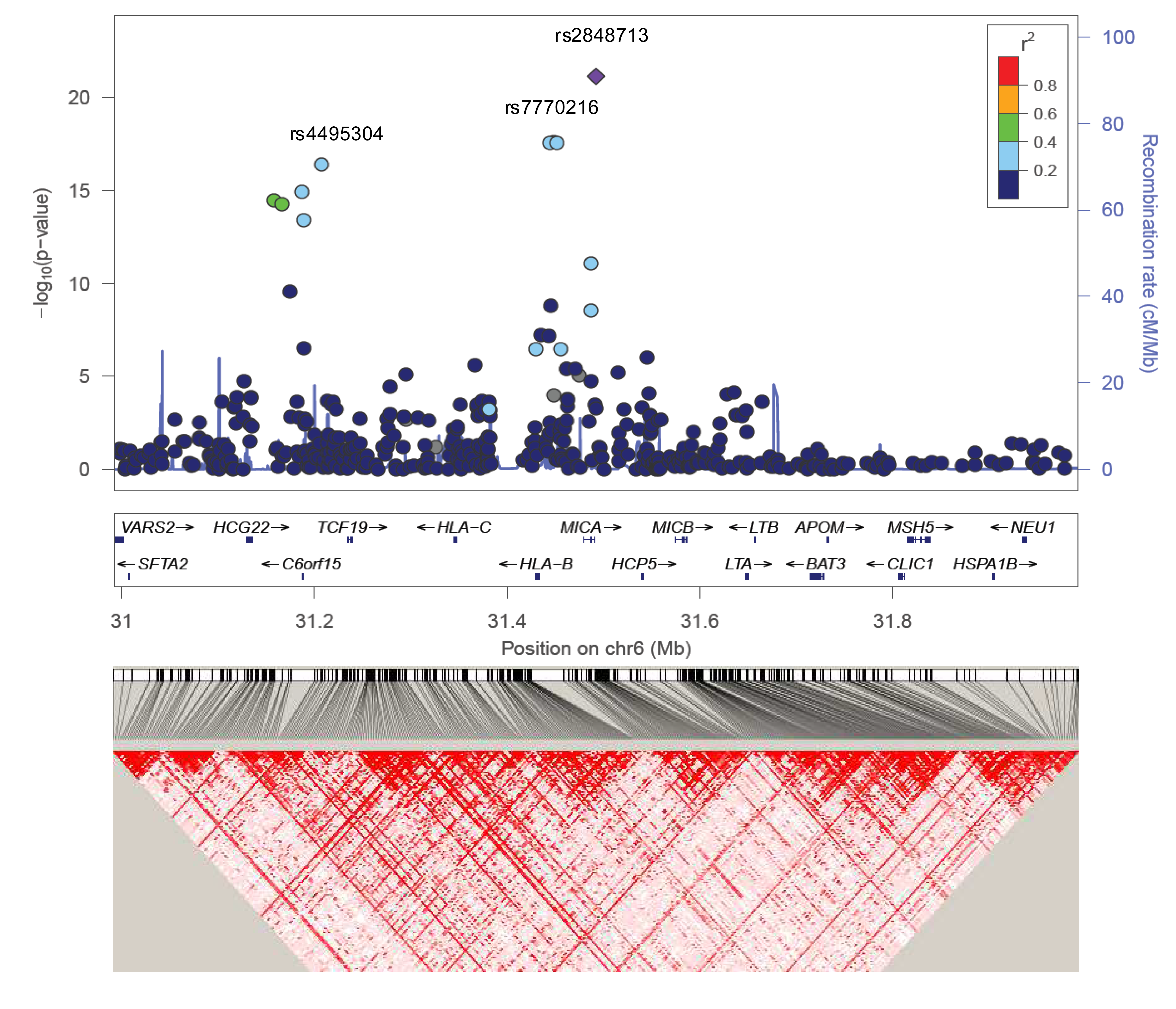

Supplement: S2 Fig — GWAS results forBehcet susceptibility using Linear Mixed Models approach. Top SNP rs2848713 EMMAX approach (MAF = 0.10, A-risk allele) is denoted by a diamond. Marker rs7770216 is the top SNP for the Genomic Principal Components adjustment approach (MAF = 0.25, T-risk allele). Marker rs4495304 is the top SNP in the association reported by Mizuki et al. (MAF = 0.09, A-risk allele) for the GWAS of Behçet susceptibility. Bottom, LD heat map based on D’ values from the combined population under study including all SNPs in the 500Kb region. (TIF) [file pone.0119085.s002.tif]
